# Renal Colic—Berberis Vulg

**Published:** 1891-11

**Authors:** S. W. Cohen

**Affiliations:** Waco, Texas


### RENAL COLIC—BERBERIS VULG.

S. W. Cohen, M. D., Waco, Texas.

A little seven year-old miss called at my residence at eight o'clock, A. M., June 21st, 1891, with these words: "Mamma says please come right away; papa can't stand it no longer." Arriving at the patient's residence, I found him walking rapidly to and fro, crying, lamenting, groaning, screaming, and throwing his hands wildly about, resting for a moment, and then again resuming his hurried walk with his hand over the region of his hip, and occasionally pressing the inguinal region. He was perspiring profusely. He was attacked at about seven o'clock that morning by a "severe cutting, screwing, and torturing pain" in the region covering the right kidney and hip. He threshed about the bed awhile, but was compelled to arise and move about, though the motion did not appear to afford any relief. He had taken Antipyrin, and as his bowels, which he suspected were the chief seat of his pains, would not move, he had taken a number of both hot water and also Glycerine enemas. His bowels always move normally, and as the enemas had no effect, he was confirmed in his opinion that his bowels were chargeable with his trouble. He had vomited once that morning and was inclined to do so constantly. The pain began in the right kidney, passed over and around the anterior superior spinous process of the ilium, thence to the inguinal and hypogastric regions.

The diagnosis was clear, but not so the indicated remedy. I had the patient placed in hot water, for I was not sure of my remedy. The water afforded some relief and felt grateful. Owing to the peculiarity of the pain, its location on the right side, and the relief obtained by heat, Magnesia-phos.$^{cc}$ was prescribed, to be administered every fifteen minutes. The pain now left the hip region and centered in the hypogastrium. The pains, though somewhat mitigated, caused the patient much uneasiness, and he would not remain quiet one moment, lamenting and moaning. I was called away and promised to return speedily. During my absence from the patient's side, I consulted my books, and concluded that Berb-vulg. was *the simillimum.* I also referred to a case cured by Dr. Sherbino with a single dose of Berberis CM, as reported by Dr. Stiles in the *Southern Journal of Homœopathy* for September, 1888. The symptoms of the two cases were very much alike, though my patient had no urinal symptoms, in fact, had not urinated that morning, nor had he any desire so to do. While still conning my books, I was hurriedly recalled to the case, and found the patient suffering more than even at any time before during the attack. He begged for Morphine incessantly, while rolling over and over on the bed. I had taken my case vial of Berberis$^{cm}$ (F.) with me, and placed a few pellets upon the patient's tongue, though with difficulty, as he was "yelling bloody murder," and going through one prolonged contortion act. Sac-lac. was prepared in water, to be administered every five minutes. I sat down to await results. In less than two minutes the rolling and screaming had ceased, and in less than five minutes I went on tip-toe from the portico into the room, and found the patient lying across the bed on his face, asleep. I took my leave, with instructions to report. At four o'clock, P. M., I heard the pleasant news that "papa had slept four hours and was free from pain." At seven o'clock that evening he was still free of pain, nor has he been troubled since, and I write this three days after I made my visits.

I have seen such cases suffer for three and four days, under Morphia, and administered, too, by homœopaths—God save the mark!

Let me here remark that the indicated remedy cured the case, although I did not even *once*, think of the *pathology* of the case, during either visit.

Very irregular, I know, but the patient didn't seem to mind that.

